# Retrospective analysis of real-world treatment patterns and clinical outcomes in patients with advanced non-small cell lung cancer starting first-line systemic therapy in the United Kingdom

**DOI:** 10.1186/s12885-021-08096-w

**Published:** 2021-05-07

**Authors:** Jason Lester, Carles Escriu, Sarah Khan, Emma Hudson, Talal Mansy, Andrew Conn, Samuel Chan, Ceri Powell, Juliet Brock, John Conibear, Lauren Nelless, Vaneet Nayar, Xiaohui Zhuo, Adeline Durand, Amerah Amin, Peter Martin, Xinke Zhang, Vivek Pawar

**Affiliations:** 1 Swansea Bay University Health Board, Port Talbot, UK; 2 Clatterbridge Cancer Centre, Wirral, UK; 3 Nottingham University Hospitals NHS Trust, Nottingham, UK; 4 Velindre University NHS Trust, Cardiff, UK; 5 South Tees Hospitals NHS Foundation Trust, Middlesbrough, UK; 6 Bradford Teaching Hospitals NHS Foundation Trust, Bradford, UK; 7 York Teaching Hospital NHS Foundation Trust, York, UK; 8 Brighton and Sussex University Hospitals NHS Trust, Brighton, UK; 9 Barts Health NHS Trust, London, UK; 10 SVMPharma Ltd., Hampshire, UK; 11 EMD Serono Research & Development Institute, Inc, Billerica, MA, USA, an affiliate of Merck KGaA, Darmstad, Germany; 12 Merck Serono Ltd, Feltham, UK, an affiliate of Merck KGaA, Darmstadt, Germany

**Keywords:** Advanced or metastatic non-small cell lung cancer, Real-world outcomes, Real-world treatment patterns

## Abstract

**Background:**

The treatment landscape for advanced non-small cell lung cancer (aNSCLC) has evolved rapidly since immuno-oncology (IO) therapies were introduced. This study used recent data to assess real-world treatment patterns and clinical outcomes in aNSCLC in the United Kingdom.

**Methods:**

Electronic prescribing records of treatment-naive patients starting first-line (1 L) treatment for aNSCLC between June 2016 and March 2018 (follow-up until December 2018) in the United Kingdom were assessed retrospectively. Patient characteristics and treatment patterns were analyzed descriptively. Outcomes assessed included overall survival (OS), time to treatment discontinuation, time to next treatment, and real-world tumor response.

**Results:**

In all, 1003 patients were evaluated (median age, 68 years [range, 28–93 years]; 53.9% male). Use of 1 L IO monotherapy (0–25.9%) and targeted therapy (11.8–15.9%) increased during the study period, but chemotherapy remained the most common 1 L treatment at all time points (88.2–58.2%). Median OS was 9.5 months (95% CI, 8.8–10.7 months) for all patients, 8.1 months (95% CI, 7.4–8.9 months) with chemotherapy, 14.0 months (95% CI, 10.7–20.6 months) with IO monotherapy, and 20.2 months (95% CI, 16.0–30.5 months) with targeted therapy. In the 28.6% of patients who received second-line treatment, IO monotherapy was the most common drug class (used in 51.6%).

**Conclusions:**

Although use of 1 L IO monotherapy for aNSCLC increased in the United Kingdom during the study period, most patients received 1 L chemotherapy. An OS benefit for first-line IO monotherapy vs chemotherapy was observed but was numerically smaller than that reported in clinical trials. Targeted therapy was associated with the longest OS, highlighting the need for improved treatment options for tumors lacking targetable mutations.

**Supplementary Information:**

The online version contains supplementary material available at 10.1186/s12885-021-08096-w.

## Background

Lung cancer is the leading cause of cancer-related death in the United Kingdom [1]. Non-small cell lung cancer (NSCLC) accounts for 80–85% of lung cancers in the United Kingdom [2], and most patients have advanced disease at initial diagnosis [3]. The use of chemotherapy to treat advanced NSCLC (aNSCLC) has increased steadily over time [4]. Platinum-based doublet therapy remains a first-line (1 L) standard of care, although it provides modest overall survival (OS) benefit and is associated with significant toxicity [5–7]. In the United Kingdom, the 1-year OS rate in patients diagnosed with aNSCLC is approximately 19%, and rates have improved very little over the past 40 years [8].

In a subgroup of aNSCLC, treatment paradigms changed dramatically with the advent of targeted therapies that inhibit oncogenic drivers, namely molecular alterations in genes encoding epidermal growth factor receptor (*EGFR*), anaplastic lymphoma kinase (*ALK*), and ROS proto-oncogene 1 (*ROS1*) [9]. Treatment options further improved with the development of immuno-oncology (IO) therapies that can activate antitumor immune responses by blocking the interaction between programmed cell death protein 1 (PD-1) and its ligand (PD-L1), which showed prolonged overall survival vs chemotherapy [10–12]. In the United Kingdom, the IO monotherapy pembrolizumab was made initially available through Early Access to Medicines Schemes; from 10 March 2016 to 31 January 2017 as a 1 L treatment, and from 10 March 2016 to 29 July 2016 as a second-line (2 L) treatment [13]. Subsequently, and similarly to other countries, IO monotherapy for aNSCLC then received positive recommendations (from the UK National Institute for Health and Care Excellence) as a 2 L treatment, starting with pembrolizumab in January 2017 and followed by nivolumab and atezolizumab in November 2017 and May 2018, respectively [14–16]. Following the publication of the UK National Institute for Health and Care Excellence (NICE) appraisal for 1 L pembrolizumab monotherapy for aNSCLC in July 2018, UK guidelines for 1 L treatment were updated to recommend that patients whose tumors had ≥50% PD-L1 expression and did not harbor molecular alterations in *EGFR* or *ALK* should receive IO monotherapy (pembrolizumab); targeted agents were recommended for patients whose tumors tested positive for *EFGR*, *ALK* and *ROS1* alterations, and platinum-based chemotherapy was recommended for other patients [17]. In June 2019, NICE approved 1 L IO therapies in combination with chemotherapy, irrespective of PD-L1 status [18–20].

Data gathered from real-world investigations can complement findings from randomized clinical trials and provide an overview of treatment patterns and outcomes in clinical practice [21]. In a systematic review of real-world studies in aNSCLC published between 2010 and 2017, including various European studies, chemotherapy was found to be the most common treatment in the 1 L, 2 L, and third-line (3 L) treatment settings [22]. In addition, a UK-based study analyzed all patients diagnosed with NSCLC but did not focus on treatment [23]. However, only limited data are available to assess the impact of IO therapy on treatment patterns in aNSCLC.

Here, we report findings from a real-world study of treatment patterns and outcomes in UK patients with aNSCLC who received 1 L treatment during the period when IO monotherapy was introduced. By using a representative network of UK treatment centers, we aimed to analyze data from approximately 10% of the annual incident population of patients with aNSCLC who were initiated on 1 L treatment (**Supplemental Figure** **1**).

## Methods

### Aim, study design and setting, and data sources

This was a retrospective, observational study of treatment-naive adults with aNSCLC who initiated 1 L systemic anticancer therapy in the United Kingdom between June 1, 2016 and March 31, 2018, and who were followed until December 31, 2018. Identified patients were included sequentially from March 31, 2018, backward. Inclusion of 1000 patients from 10 sites was planned. Initial patient data were obtained via database abstraction from electronic prescribing records, which are widely used within hospitals in the UK and record all anticancer prescriptions dispensed to patients, including physician defined treatment lines and cycles. Subsequently, electronic prescribing record data from provisionally eligible patients were entered into electronic case report forms, which were sent to hospitals for supplementation using patient notes. On the basis of available information and the eligibility criteria below, patients were included or excluded from the final analysis. Data was pseudonymized at source; hence, patient consent was not required.

### Eligibility criteria

Patients were included if they were aged ≥18 years and were diagnosed with advanced or metastatic NSCLC at the time of initiating 1 L systemic anticancer treatment and had received no prior treatment for aNSCLC. Advanced disease was defined as ≥1 of the following: (1) physician-defined stage IV disease, (2) TNM staging with an M value of 1, (3) patient record identifying the location of metastatic disease, or (4) current or prior disease status containing a reference to advanced or metastatic disease. Patients were excluded if they had been enrolled in a clinical trial at any time during the study period or if required study data were missing.

### Statistical analysis

Patient and disease characteristics were collected at diagnosis and analyzed descriptively. Outcomes analyzed were: OS (interval between 1 L treatment initiation and date of death from any cause); time to treatment discontinuation (TTD; interval between 1 L treatment initiation and discontinuation for any reason, including death), which provides an indication of both progression-free survival and tolerability (ie, discontinuation due to progression or toxicity); time to next treatment (TtNT; interval between 1 L treatment initiation and 2 L treatment initiation or death), assessed to determine any benefit in treatment-free interval; and real-world tumor response (rwTR; physician-defined best response of either partial or complete response), which was analyzed as a surrogate for objective response rate [24]. Time to event outcomes (OS, TTD, and TtNT) were analyzed using the Kaplan-Meier method; patients who were event-free during the study observation period were censored on their last assessment date or at the study end date (whichever occurred first). For the analysis of TTD, in patients who discontinued treatment but were still alive, the treatment end date was recorded as the start date of the last treatment cycle because a definitive end date of the last cycle was not available, and the last cycle start date was the latest date when it was certain that treatment was continuing.

Data for baseline characteristics and patient outcomes were analyzed in the overall population and in discrete subgroups defined by 1 L drug class, ie, IO monotherapy (anti–PD-1 or PD-L1 antibody), targeted therapy (inhibitor of EGFR, ALK, or ROS1), or chemotherapy (cytotoxics and other agents).

Analysis of treatment patterns included overall breakdown by drug class and regimen, overall treatment sequencing by drug class, and change in 1 L use of drug classes over time. A regimen included all drugs used in each line of treatment.

## Results

### Patients

Of 1257 patients who were assessed for eligibility, 1003 treatment-naive patients from 9 sites (**Supplemental Table** **1**) who initiated 1 L therapy for aNSCLC between June 1, 2016, and March 31, 2018, were included in the study population (Table 1). Patient data were not obtained from 1 of 10 planned sites because of capacity issues; 2 sites (Nottingham University Hospitals NHS Trust and the Clatterbridge Cancer Centre NHS Foundation Trust) contributed 56% of patients. In the study population, the median age was 68 years (range, 28–93 years), 53.9% were male, Eastern Cooperative Oncology Group performance status score was 0–1 in 75.7% and ≥ 2 in 24.3%, and tumor histology was non-squamous in 63.9%, squamous in 24.2%, and unknown in 11.9% (Table 2). All patients had metastatic disease at diagnosis.

**Table 1:** Summary of Patient Numbers in the Study Population and Reasons for Exclusion (Study Attrition)

	Patients	
	*n*	%
**Adults who received 1 L treatment for NSCLC**	**1257**	**–**
**Excluded patients**	**254**	**100**
NSCLC not advanced or metastatic at diagnosis	151	59.4
Treatment started outside of study period	47	18.5
ECOG PS score missing	15	5.9
Randomized trial participant	9	3.5
Response data missing	8	3.1
Never received treatment	7	2.8
Duplicate patient	5	2.0
Age missing	4	1.6
Sex missing	3	1.2
Not NSCLC	2	0.8
Date of death or last hospital follow-up missing	1	0.4
Histological diagnosis missing	1	0.4
Diagnosis date unknown	1	0.4
**Met inclusion criteria**	**1003**	**–**

*1 L* first line; *ECOG PS* Eastern Cooperative Oncology Group performance status; *NSCLC* non-small cell lung cancer

**Table 2:** Patient Demographics in the Overall Population and in Subgroups Defined by 1 L Drug Class Received

	All patients (***n*** = 1003)	1 L chemotherapy (***n*** = 698)	1 L IO monotherapy (***n*** = 179)	1 L targeted therapy (***n*** = 126)
**Proportion of study population, %**	100	69.6	17.8	12.6
**Median follow-up (range), months**	9.2 (0.0–42.7)	7.9 (0.0–42.7)	12.7 (0.1–37.3)	16.3 (0.1–37.1)
**Median age at diagnosis (range), years**	68 (28–93)	68 (28–88)	67 (48–90)	70 (32–93)
**Sex, n (%)**				
Male	541 (53.9)	395 (56.6)	94 (52.5)	52 (41.3)
Female	462 (46.1)	303 (43.4)	85 (47.5)	74 (58.7)
**Tumor histology, n (%)**				
Adenocarcinoma	635 (63.3)	387 (55.4)	131 (73.2)	117 (92.9)
Squamous cell carcinoma	243 (24.2)	202 (28.9)	38 (21.2)	3 (2.4)
Large cell carcinoma	6 (0.6)	4 (0.6)	2 (1.1)	0
Not specified	119 (11.9)	105 (15.0)	8 (4.5)	6 (4.8)
**TNM stage at diagnosis, n (%)**				
T				
T X-4	938 (93.5)	647 (92.7)	170 (95.0)	121 (96.0)
N/A	65 (6.5)	51 (7.3)	9 (5.0)	5 (4.0)
N				
N X-3	939 (93.6)	648 (92.8)	170 (95.0)	121 (96.0)
N/A	64 (6.4)	50 (7.2)	9 (5.0)	5 (4.0)
M				
M1^a^	524 (52.2)	351 (50.3)	114 (63.7)	59 (46.8)
M1a	166 (16.6)	120 (17.2)	22 (12.3)	24 (19.0)
M1b	310 (30.9)	224 (32.1)	43 (24.0)	43 (34.1)
M1c	3 (0.3)	3 (0.4)	0	0
**ECOG PS score at diagnosis, n (%)**				
0–1	759 (75.7)	513 (73.5)	157 (87.7)	89 (70.6)
2+	244 (24.3)	185 (26.5)	22 (12.3)	37 (29.4)
***EGFR*+ status, n (%)**^b^				
Documented	19 (1.9)	1 (0.1)	0	18 (14.3)
Assumed	89 (8.9)	0	0	89 (70.6)
***ALK*+ status, n (%)**^b^				
Documented	2 (0.2)	0	0	2 (1.6)
Assumed	17 (1.7)	0	0	17 (13.5)
**PD-L1+ status, n (%)**^b^				
Documented	10 (1.0)	3 (0.4)	7 (3.9)	0
Assumed	172 (17.1)	0	172 (96.1)	0

*1 L* first line; *ALK* anaplastic lymphoma kinase; *ECOG PS* Eastern Cooperative Oncology Group performance status; *EGFR* epidermal growth factor receptor; *IO* immuno-oncology; *NSCLC* non-small cell lung cancer; *PD-L1* programmed death-ligand 1

^a^ Includes 77 patients with clinician-defined stage IV NSCLC. ^b^ Biomarker status was based either on hospital test results (documented) or treatment regimen (assumed, ie, patients who received an EGFR or ALK inhibitor were assumed to have a tumor harboring an *EGFR* or *ALK* mutation, and patients receiving IO therapy were assumed to have a PD-L1+ tumor)

### Treatment patterns and sequencing

**Fig. 1:**
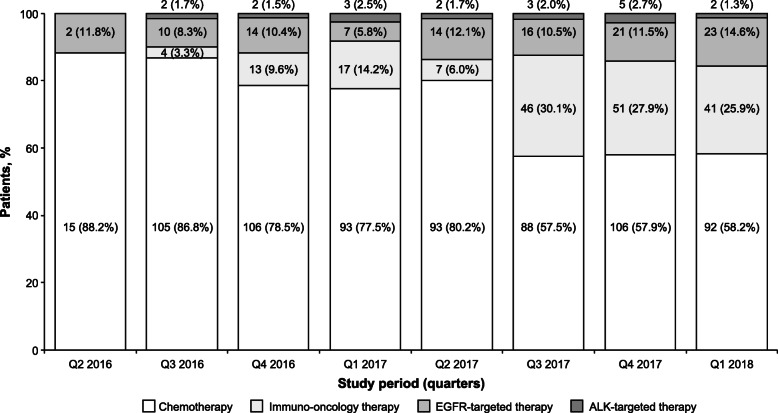
Quarterly Change in Proportions of First-line Use for Each Drug Class Over Study Observation Period. ALK, anaplastic lymphoma kinase; EGFR, epidermal growth factor receptor; Q, quarter

First-line treatment comprised chemotherapy in 698 patients (69.6%), IO monotherapy in 179 patients (17.8%), and targeted therapy in 126 patients (12.6%). Among chemotherapy-treated patients, 674 (96.6%) received platinum-based chemotherapy, and carboplatin-based doublet or triplet chemotherapy was the most commonly administered regimen (*n* = 499 [71.5% of chemotherapy-treated patients]; Table 3). Among patients who received 1 L IO monotherapy or targeted therapy, pembrolizumab (*n* = 174 [97.2% of the 1 L IO subgroup]) and afatinib (*n* = 67 [53.2% of the 1 L targeted therapy subgroup]) were the most commonly administered agents, respectively. During the time period analyzed (June 2016 to March 2018), the proportions of patients receiving 1 L IO monotherapy or targeted therapy increased (from 0 to 25.9% for IO therapy, and from 11.8 to 15.9% for targeted therapy), whereas the proportion of 1 L chemotherapy-treated patients decreased (from 88.2 to 58.2%; Fig. 1).

**Table 3:** First-Line and Second-Line Treatment Regimens

Regimen	Patients, n (%)	
	1 L therapy (*n* = 1003)	2 L therapy (***n*** = 287)
**Chemotherapy**		
Carboplatin-based doublet or triplet therapy^a^	499 (49.8)	57 (19.9)
Carboplatin	3 (0.3)	0
Cisplatin-based doublet or triplet therapy^a^	172 (17.1)	7 (2.4)
Docetaxel	5 (0.5)	17 (5.9)
Docetaxel + nintedanib	4 (0.4)	16 (5.6)
Gemcitabine	3 (0.3)	1 (0.3)
Nintedanib	0	1 (0.3)
Paclitaxel	0	4 (1.4)
Pemetrexed	9 (0.9)	0
Vinorelbine	3 (0.3)	1 (0.3)
**Immuno-oncology therapy**		
Atezolizumab	0	32 (11.1)
Nivolumab	5 (0.5)	20 (7.0)
Pembrolizumab	174 (17.3)	96 (33.4)
**Targeted therapy**^b^		
Afatinib	67 (6.7)	5 (1.7)
Alectinib	2 (0.2)	2 (0.7)
Ceritinib	6 (0.6)	3 (1.0)
Crizotinib	11 (1.1)	4 (1.4)
Erlotinib	12 (1.2)	5 (1.7)
Gefitinib	24 (2.4)	3 (1.0)
Osimertinib	4 (0.4)	13 (4.5)

*1 L* first line; *2 L* second line; *ALK* anaplastic lymphoma kinase; EGFR, epidermal growth factor receptor;* IV *intravenous,* ROS* ROS proto-oncogene 1

^a^ Triplet therapy indicates carboplatin (IV) + vinorelbine (IV) + vinorelbine (oral), cisplatin (IV) + vinorelbine (IV) + vinorelbine (oral), or cisplatin (IV) + etoposide (IV) + etoposide (oral)

^b^ EGFR, ALK, or ROS inhibitor

In the overall population, 287 patients (28.6%) received 2 L therapy, which was chemotherapy in 104 (36.2%), IO monotherapy in 148 (51.6%), and targeted therapy in 35 (12.2%; Table 3). The most common 2 L therapy was pembrolizumab (*n* = 96 [33.4%]). The most common treatment sequence was 1 L chemotherapy, followed by 2 L IO monotherapy (*n* = 146 [20.9% of those who received 1 L chemotherapy]) or 2 L chemotherapy (*n* = 74 [10.6% of those who received 1 L chemotherapy]; **Supplemental Figure** **2**). Of patients who had received 1 L IO monotherapy, chemotherapy was the most common 2 L treatment (*n* = 26 [14.5% of those who received 1 L IO monotherapy]). Of patients who received 1 L targeted therapy, targeted therapy was also the most commonly used 2 L treatment class (n = 26 [20.6% of those who received 1 L targeted therapy]).

Of 716 patients who did not receive 2 L therapy, 77.0% died (88.7, 56.3, and 52.1% of those who had received 1 L chemotherapy, IO monotherapy, or targeted therapy, respectively), 12.8% had ongoing 1 L treatment (1.9, 31.8, and 36.5% of those who received 1 L chemotherapy, IO monotherapy, or targeted therapy, respectively), and 10.2% stopped treatment and were still alive at the end of the study period (9.4, 11.9, and 11.5% of those who had received 1 L chemotherapy, IO monotherapy, or targeted therapy, respectively; **Supplemental Table** **2**).

Of the 287 patients who received 2 L treatment, 51 subsequently received 3 L treatment (5.1% of the total population or 17.8% of the 2 L population [21.3%, excluding 47 patients who continued to receive 2 L therapy at last follow-up]). Eleven patients received fourth-line (4 L) therapy (1.1% of the total population or 21.6% of the 3 L population [28.2%, excluding 12 patients who continued to receive 3 L therapy at last follow-up]), and 1 patient received fifth-line therapy (0.1% of the total population or 9.1% of the 4 L population [10.0%, excluding 1 patient who continued to receive 4 L therapy at last follow-up]; **Supplemental Figure** **2**).

### Clinical outcomes

**Fig. 2:**
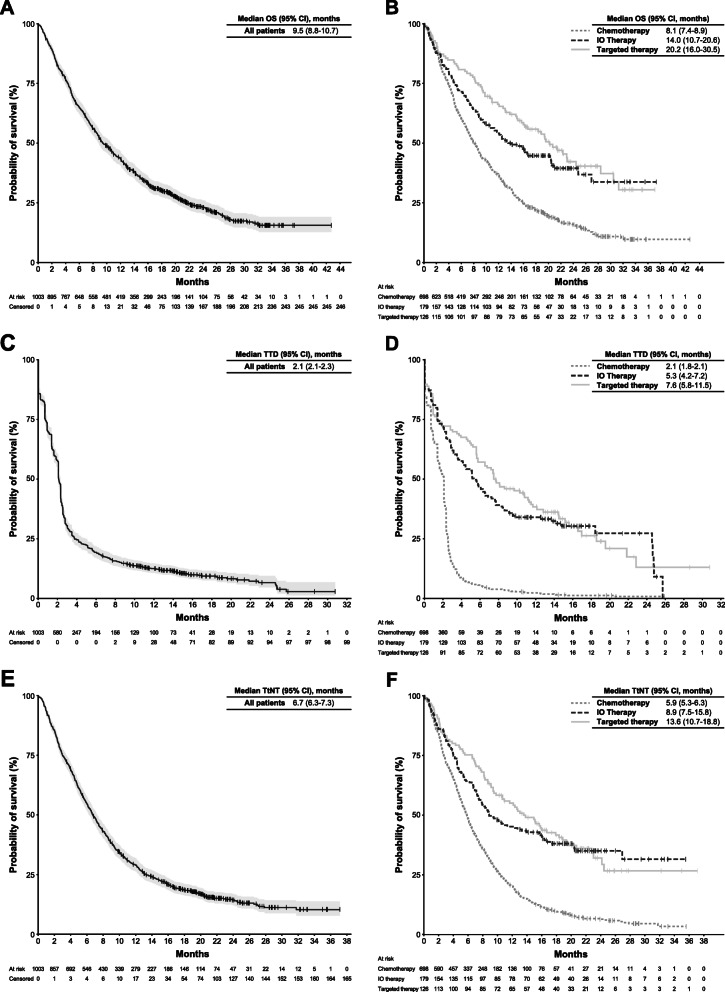
Clinical Outcomes. Kaplan-Meier Plots of **a** overall survival (OS) in the overall population, **b** OS by first-line (1 L) drug class, **c** time to treatment discontinuation (TTD) in the overall population, **d** TTD by 1 L drug class, **e** time to next therapy (TtNT) in the overall population, and **f** TtNT by drug class. Dashed lines denote median values. IO, immuno-oncology

In the overall population, the median follow-up was 9.2 months (95% CI, 0–42.7 months), with a longer median follow-up in the IO monotherapy group (12.7 months, 95% CI, 0.1–37.3 months) and targeted therapy group (16.3 months, 95% CI, 0.1–37.1 months), and a shorter median follow-up in the chemotherapy group (7.9 months, 95% CI, 0–42.7 months) (Table 2). The median OS was 9.5 months (95% CI, 8.8–10.7 months; Fig. 2a) in the entire population. Within 1 L subgroups defined by drug class, median OS was longest in patients who had received 1 L targeted therapy (median 20.2 months [95% CI, 16.0–30.5 months]), followed by patients who had received 1 L IO monotherapy (median 14.0 months [95% CI, 10.7–20.6 months]), and was shortest in patients who had received 1 L chemotherapy (median 8.1 months [95% CI, 7.4–8.9 months]; Fig. 2b). In the overall population, median TTD from 1 L was 2.1 months (95% CI, 2.1–2.3 months; Fig. 2c). Within subgroups, the median TTD was longest with 1 L targeted therapy (median, 7.6 months [95% CI, 5.8–11.5 months]) and was 5.3 months (95% CI, 4.2–7.2 months) with 1 L IO monotherapy and 2.1 months (95% CI, 1.8–2.1 months) with 1 L chemotherapy (Fig. 2d). Median TtNT from 1 L was 6.7 months (95% CI, 6.3–7.3 months) in the overall study population (Fig. 2e); in 1 L subgroups, it was 13.6 months (95% CI, 10.7–18.8 months) with 1 L targeted therapy, 8.9 months (95% CI, 7.5–15.8 months) with 1 L IO monotherapy, and 5.9 months (95% CI, 5.3–6.3 months) with 1 L chemotherapy (Fig. 2f).

In the overall population, 291 patients (29.0%) had an rwTR. Within 1 L subgroups, rwTRs occurred in 187 (26.8%) of those who received 1 L chemotherapy, 61 (34.1%) of those who received 1 L IO monotherapy, and 43 (34.1%) of those who received 1 L targeted therapy.

## Discussion

In this retrospective study, we assessed real-world treatment patterns and outcomes in treatment-naive patients with aNSCLC who started 1 L systemic anticancer therapy in the United Kingdom between June 2016 and March 2018. To our knowledge, this is the first large-scale UK study to report data in this setting. By obtaining data from more than 1000 patients treated at both tertiary cancer centers and district general hospitals, this study provides a robust and generalizable dataset describing real-world 1 L treatment for aNSCLC. Patient characteristics in our study population are comparable to previous real-world UK studies in NSCLC [25, 26]. Our findings show that IO monotherapy has been adopted rapidly as 1 L therapy following reimbursement approval in the United Kingdom, and although use of 1 L chemotherapy decreased over the study period, chemotherapy remained the most common 1 L treatment. IO therapy was the most commonly used 2 L treatment, possibly reflecting the greater length of time these agents have been available to UK patients, the increased availability of IO agents in 2 L and wider eligibility criteria and the fact that 2 L chemotherapy is associated with modest benefit but substantial toxicity.

PD-L1 biomarker information was not recorded in our dataset; thus, it was not possible to assess the use of 1 L IO monotherapy or chemotherapy with respect to PD-L1–positive status. It has been estimated previously that 23–28% of patients with aNSCLC have tumor cell PD-L1 expression ≥50% [27, 28]. Given that 1 L IO monotherapy usage in our analysis increased from 0 to 25.9% during study follow-up, it appears that IO monotherapy may have been used in the vast majority of patients whose cancers have PD-L1 expression of ≥50%. Furthermore, excluding patients whose tumors harbored *EGFR* or *ALK* mutations (who are ineligible for pembrolizumab within the approved indication), 1 L IO monotherapy was received in the final full quarter by 30.8% (41/133) of all patients, which is consistent with the incidence of PD-L1 tumor proportion score ≥ 50% reported in the KEYNOTE-024 trial (30.2% of all screened patients with PD-L1 status) in this population [7].

Only around 30% of patients in our study population received 2 L therapy, which emphasizes the importance of selecting the most effective 1 L treatment. This observation may reflect the poor condition of patients with disease progression after 1 L treatment and/or perceptions of the risk-benefit ratio for 2 L treatment options in the population. This pattern of low proportion of patients receiving 2 L treatment was also documented in a study by Nadler et al. investigating the real-world treatment patterns and outcomes using data in the US community setting [29].

Despite the increase in 1 L treatment options for aNSCLC in recent years, patient outcomes remained poor in our study population, with a median OS of only 9.5 months, median TTD of 2.1 months, and rwTR in 29.0%. IO monotherapy was associated with improved patient outcomes, with a median OS of 14.0 months, median TTD of 5.3 months, and rwTR in 34.1%, supporting the clinical benefits reported in clinical trials when compared with chemotherapy [10–12]. OS and TTD were longest with targeted therapy (median 20.2 and 7.6 months, respectively), with rwTR rates identical to 1 L IO monotherapy (34.1%). Overall, these data illustrate that more effective treatments are needed, particularly for patients who are ineligible for targeted therapy.

OS with 1 L IO monotherapy in this real-world study was shorter than has been reported in clinical trials. For example, the median OS with 1 L IO monotherapy was 14.0 months, compared with 26.3 months in the KEYNOTE-024 trial of 1 L pembrolizumab [30]. In addition, median TTD and median TTNT with 1 L IO monotherapy in our study were 5.3 months and 8.9 respectively, whereas the median PFS with pembrolizumab in KEYNOTE-024 was 10.3 months [10], although it should be noted that TTD captures discontinuations for all reasons, including progressive disease and toxicity. The shorter outcomes in this real-world study compared with randomized controlled trials should be interpreted with caution because of the differences between the heterogeneous population of patients treated in a clinical practice and the highly selected, “favorable-risk” populations eligible for clinical trials. For example, 24.3% of our population had an Eastern Cooperative Oncology Group performance status of ≥2, whereas these patients are typically excluded from oncology trials. Similarly, the rwTR rate for IO monotherapy in our study was lower than the objective response rate of 44.8% of pembrolizumab reported in the KEYNOTE-24 trial [10]. In addition to the patient heterogeneity between real-world studies and clinical trials, it is important to note that the rwTR was based on physician’s notes in patient records rather than Response Evaluation Criteria in Solid Tumors (RECIST) criteria used in clinical trials. Therefore, in the real world, response may not always be reliably and consistently documented. The patient population in our study is also different from that assessed in other recent real-world studies in NSCLC, which focused on patients who continued treatment beyond 1 L therapy [22] or included patients with early-stage NSCLC who received treatment with curative intent [31], which is not applicable to aNSCLC.

Our study has several acknowledged limitations. First, patient observation was limited with a median follow-up of 9.2 months; thus, outcome events (e.g. death for OS, treatment discontinuation for TTD) have not been observed in some patients and long-term survival data are immature. Second, only 3.1% of patients included had documented test results for oncogenic driver mutations or PD-L1 expression; thus, it was not possible to assess whether IO therapy or targeted therapy was used in the appropriate patient population as defined by clinical guidelines. For example, it is possible that PD-L1 test results were available before *EGFR* or *ALK* test results, and as a result, physicians might have initiated IO therapy in patients with high PD-L1 expression who were subsequently found to have an *EGFR* or *ALK* genetic alteration. However, among patients who received 1 L IO therapy and received 2 L treatment in our study, no patient received an EGFR or ALK inhibitor as 2 L treatment, suggesting that these patients were unlikely to have an *EGFR* mutation or *ALK* rearrangement. Similarly, although PD-L1 biomarker information was not available in our dataset, use of 1 L IO monotherapy in the United Kingdom during the study period was restricted to patients with a PD-L1 tumor proportion score of ≥50%; thus, it is likely that only patients with a PD-L1 tumor proportion score ≥ 50% received IO monotherapy in this population. Third, patients were assessed during a period when 1 L IO monotherapy was first introduced. Outcomes of patients now treated with 1 L IO monotherapy may reasonably be expected to be better than during the period of this study; patient selection, assessment of response and management of side effects have evolved as clinical experience increased.

In addition, a retrospective study such as this will inevitably provide a historical perspective on treatment pathways. Further real-world studies are needed to evaluate whether outcomes in aNSCLC have improved with the introduction of IO-based combination regimens or other novel therapies in clinical practice.

With regard to the generalizability of our results to the UK patient population, only sites with the capacity to participate were included, and 2 sites contributed more than half of patients. In addition, patients missing essential data were excluded, which may have differentially impacted hospitals with different follow-up capacities. For the analysis of TTD, the treatment end date in patients who discontinued treatment but were still alive was entered as the start date of the last treatment cycle, whereas the true treatment end date may have occurred several weeks later.

## Conclusions

In this retrospective study of patients who received treatment for aNSCLC in UK clinical practice, 1 L IO monotherapy was increasingly used between June 2016 and March 2018, but chemotherapy remained the most common 1 L treatment. A minority of patients received 2 L treatment. Patients treated with 1 L IO monotherapy had a longer overall survival compared to those treated with 1 L chemotherapy. OS was longest in patients who received 1 L targeted therapy, suggesting that improved treatment options are needed for patients with aNSCLC without *EGFR*, *ALK or ROS1* alterations.

## Supplementary Information

**Additional file 1 Supplemental Table 1.** Sites Included in the Study**Supplemental** Table 2**.** Outcomes of Patients Who Did Not Receive Second-Line Therapy. **Supplemental Figure 1.** Estimated NSCLC Population Size in England Based on 2018–2019 Data. **Supplemental Figure 2.** Treatment Sequences in the Study Population. Percentage values were calculated using the denominator of patients who received the preceding therapy in the sequence.

## Data Availability

The data controllers and owners are the hospital trusts; the authors do not have permission to disseminate this data without approval of the owners.

## Abbreviations

1 L: First line
2 L: Second line
3 L: Third line
4 L: Fourth line
ALK: Anaplastic lymphoma kinase
aNSCLC: advanced non-small cell lung cancer
EGFR: Epidermal growth factor receptor
IO: Immuno-oncology
NSCLC: Non-small cell lung cancer
OS: Overall survival
PD-1: Programmed cell death protein 1
PD-L1: Programmed death-ligand 1
ROS1: ROS proto-oncogene 1
rwTR: Real-world tumor response
TTD: Time to treatment discontinuation
TtNT: Time to next treatment
