# Supplementary material for: Retrospective analysis of real-world treatment patterns and clinical outcomes in patients with advanced non-small cell lung cancer starting first-line systemic therapy in the United Kingdom
Source: BMC Cancer. 2021 May 7;21:515. doi: 10.1186/s12885-021-08096-w (PMC8106229; doi:10.1186/s12885-021-08096-w)
Supplement: Supplementary file 1 — Additional file 1 Supplemental Table 1. Sites Included in the StudySupplemental Table 2. Outcomes of Patients Who Did Not Receive Second-Line Therapy. Supplemental Figure 1. Estimated NSCLC Population Size in England Based on 2018–2019 Data. Supplemental Figure 2. Treatment Sequences in the Study Population. Percentage values were calculated using the denominator of patients who received the preceding therapy in the sequence. [file 12885_2021_8096_MOESM1_ESM.docx]

# SUPPLEMENTAL DATA

**Supplemental Table 1. Sites Included in the Study**

| Site | Investigator(s) |
| --- | --- |
| Barts Health NHS Trust, London | John Conibear |
| Bradford Royal Infirmary, Bradford Teaching Hospitals NHS Foundation Trust, Bradford | Andrew Conn |
| Brighton and Sussex University Hospitals NHS Trust, Brighton and Haywards Heath | Juliet Brock |
| The Clatterbridge Cancer Centre NHS Foundation Trust, Bebington, Wirral | Carles Escriu |
| Nottingham University Hospitals NHS Trust, Nottingham | Sarah Khan |
| Singleton Hospital, Swansea Bay University Health Board, Port Talbot | Jason Lester, Ceri Powell |
| South Tees Hospitals NHS Foundation Trust, Middlesbrough | Talal Mansy |
| Velindre NHS Trust, Cardiff | Emma Hudson |
| York Teaching Hospital NHS Foundation Trust, York | Samuel Chan |

NHS, UK National Health Service.

**Supplemental Table 2. Outcomes of Patients Who Did Not Receive Second-Line Therapy**

| **n (%)** | **Any first-line therapy  (n=716)** | **First-line therapy subgroup** | | |
| --- | --- | --- | --- | --- |
|  |  | **Chemotherapy (n=469)** | **Immuno-oncology monotherapy (n=151)** | **Targeted therapy (n=96)** |
| **Ongoing treatment** | 92 (12.8) | 9 (1.9) | 48 (31.8) | 35 (36.5) |
| **Stopped treatment (alive)** | 73 (10.2) | 44 (9.4) | 18 (11.9) | 11 (11.5) |
| **Died** | 551 (77.0) | 416 (88.7) | 85 (56.3) | 50 (52.1) |

**Supplemental Figure 1. Estimated NSCLC Population Size in England Based on 2018-2019 Data**

1L, first-line; 2L, second-line; 3L, third-line; ALK, anaplastic lymphoma kinase; EGFR, epidermal growth factor receptor; NSCLC, non-small-cell lung cancer; PD-L1, programmed death-ligand 1.

Source for estimated population sizes in each step:

National Institute for Health and Care Excellence. Resource impact report: Pembrolizumab for treating PD-L1-positive non-small-cell lung cancer after chemotherapy (TA428). September 2017. Available at <https://www.nice.org.uk/guidance/ta428/resources/resource-impact-report-pdf-4355528077>. Accessed May 20, 2020.

**Supplemental Figure 2. Treatment Sequences in the Study Population.** Percentage values were calculated using the denominator of patients who received the preceding therapy in the sequence


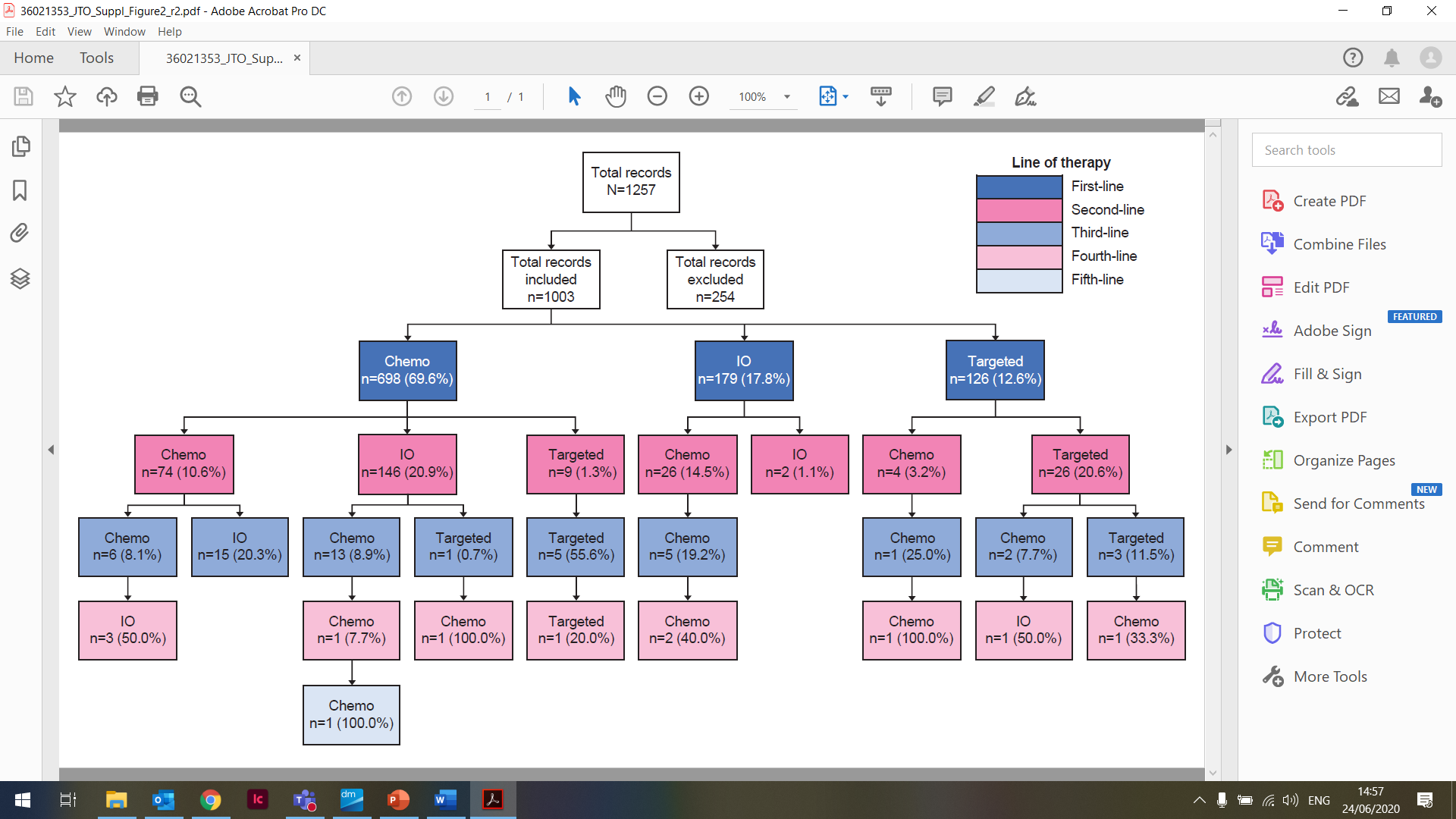


Chemo, chemotherapy; IO, immuno-oncology monotherapy; Targeted, targeted therapy.
